# Existence and perceived application of pain management protocols in German neonatal intensive care units

**DOI:** 10.1002/pne2.12089

**Published:** 2022-10-05

**Authors:** Melissa Ulmer, Kyriakos Martakis, Nadine Scholten, Ludwig Kuntz

**Affiliations:** ^1^ Department of Business Administration and Health Care Management, Faculty of Management, Economics and Social Sciences University of Cologne Cologne Germany; ^2^ Department of Pediatrics, University Hospital, Faculty of Medicine University of Cologne Cologne Germany; ^3^ Department of Pediatric Neurology, University Children's Hospital (UKGM), Faculty of Medicine Justus Liebig University of Giessen Cologne Germany; ^4^ Institute of Medical Sociology, Health Services Research and Rehabilitation Science, Faculty of Human Sciences and Faculty of Medicine University of Cologne Cologne Germany

**Keywords:** Germany, neonatal intensive care units, nurses, pain management, physicians, standard operating procedures

## Abstract

We explored the existence and application of standard operating procedures (SOPs) for pain management (PM) in German neonatal intensive care units (NICUs), and identified the factors associated with their application in practice. This study was part of the Safety4NICU project, a cross‐sectional survey conducted from 2015 to 2016. All 224 German NICUs were invited to participate, providing written consent from the head neonatologist and head nurse. We distributed questionnaires to the head neonatologist, the head nurse, and the NICU staff (physicians and nurses). We asked the head neonatologist whether written SOPs for PM existed, and we asked the staff whether these SOPs were applied in their daily routine. We received evaluable responses from 468 physicians and 1251 nurses from 76 NICUs. Of these 76 NICUs, the head neonatologists from 54 NICUs (71.1%) reported that written SOPs for PM exist. However, only 48.5% of the physicians and 53.7% of the nurses declared that these existing SOPs were also applied. We found various predictors for the existing SOPs as being applied, depending on the profession. For physicians, clinical training was important (OR: 2.482, *p* ≤ 0.05), while for nurses their working experience was a decisive predictor (OR: 1.265, *p* ≤ 0.05). For both, a high level of perceived cooperative norms between physicians and nurses increased the probability that SOPs for PM were applied, whereas a high bed turnover rate decreased that probability. According to the responses from head neonatologists, written SOPs for PM were common in German NICUs. However, if management strategies on pain existed, this did not mean that these were directly applied in the daily routine. Clinical training of the staff, the promotion of adequate interprofessional cooperation, as well as allowing time to deal with these SOPs might be all essential measures to strengthen the application.

## 1 INTRODUCTION

Premature infants are often exposed to painful diagnostic and therapeutic interventions in the context of intensive care treatment during their neonatal intensive care unit (NICU) stay. A systematic literature review of observational studies showed that the frequency of painful interventions during the NICU stay averaged 7.5 to 17.3 per neonate per day.^1^ These included insertion of peripheral catheters, heel pricks, suctioning, and venipuncture, and were often performed without providing adequate analgesic, comfort, or pain modulatory measures.^1^ Prior research revealed that early and prolonged pain exposure also led to negative long‐term consequences for these infants.^2^, ^3^, ^4^, ^5^ A large prospective cohort study in 243 NICUs in 18 European states found that pain assessment was still performed on only one‐third of the admitted infants, and on only 10% of them on a daily basis.^6^

Systematic pain assessment shall be followed by standardized management, using both pharmacological and nonpharmacological treatment that addresses anticipated or reliably recognized neonatal pain.^7^, ^8^ The use of NICU‐specific protocols or standard operating procedures (SOPs, i.e., a detailed and written instruction on how to perform a process) for pain management (PM) that include pain assessment and treatment is a crucial step in this application process.^9^ For example, the presence of guidelines in NICUs leads to a higher use of pain assessment scales^10^ and increases the perception of the necessity of pharmacological measures.^11^ The lack of standardization has often been reported as problematic among NICU staff in different NICU settings internationally.^11^, ^12^, ^13^, ^14^ If pain tools are not used at all, or not used adequately, there is a risk of undertreatment of pain and over‐medication, which might lead to avoidable adverse drug reactions.^15^

Although written guidelines for PM may exist, their application should not be taken for granted. For instance, Jeong et al.^11^ showed that 21.3% of nurses had not read their NICU's guidelines on neonatal PM. Moreover, Cong et al.^14^ revealed that only 79.3% of American nurses and 44.3% of Chinese nurses were aware of the PM guidelines in their unit.

Until now, different aspects have been suggested as being associated with the application of PM, for example, the knowledge about pain and misconceptions about the quality of comfort care and pediatric palliative care,^16^ the profession,^17^ the time of day,^18^ and the presence of parents.^1^

In previous research, training and sufficient knowledge about neonatal pain assessment were named as important factors when it came to pain assessment and management.^16^, ^19^, ^20^, ^21^ For example, the study by Carlsen Misic et al.^19^ demonstrated that 34% of nurses in neonatal units felt insecure and perceived their knowledge as insufficient with regard to using a pain scale correctly. Further, Capolingua and Gill^22^ found out that nurses with postgraduate clinical training reported more positive attitudes toward premature infant pain assessment and management. They used analgesia for painful procedures more often.

Besides the importance of clinical training, it was reported that a nurse's professional experience was positively associated with a higher knowledge of guidelines and protocols.^20^ Hamdan et al.^23^ reported that nurses with a working experience of more than 5 years read recommendations by their country's Pain Society more often.

Further, within an open questionnaire given to Swedish nurses on neonatal units, the cooperation between nurses and physicians was mentioned as a decisive factor for an adequate PM.^19^ Nurses in Polish hospitals also mentioned inadequate communication within the team as a barrier in effective PM.^20^

More aspects that were revealed as barriers in pain assessment and management were time pressure and a high workload, respectively.^23^, ^24^, ^25^

Our study followed two main aims. First, we aimed to explore and describe the existence and the application of written SOPs for PM by nurses and physicians in German NICUs. Our second aim was to analyze factors that might promote the nurses' and physicians' application of existing written SOPs for PM.

Taking into account the scientific background, we derived the following four hypotheses:

Hypothesis 1 Additional clinical training is positively associated with the application of existing SOPs for PM.

Hypothesis 2 Professional experience in the NICU is positively associated with the application of existing SOPs for PM.

Hypothesis 3 A high level of perceived cooperative norms between physicians and nurses positively affects the application of existing SOPs for PM.

Hypothesis 4 The higher the workload is that is measured by the bed turnover rate (BTR) of infants with a very low birth weight, the lower is the application of existing SOPs for PM.

## 2 METHODS

### 2.1 Study design

The present study was part of the research project “The Effect of Leadership Skills and Leaders’ Affective Configuration on Outcomes”—Safety4NICU, conducted by the University of Cologne Forum: Managerial Risk Factors in Medicine. The project was an extensive, prospective, questionnaire‐based survey in cross‐sectional design, conducted from 2015 to 2016. The study was approved by the Ethics Committee of the Faculty of Medicine of the University of Cologne and can be retrieved in the German Clinical Trials Register with the trial number DRKS00007724.

The head neonatologists and head nurses of all identified 224 German NICUs (levels of care I and II) were invited to participate in the survey. In German NICUs, the level of care I provides intensive care services to the most premature and the sickest infants, differing from the level of care II in the number of staff and the available facilities.^26^ The invited NICUs were identified by public reports and web‐based search. Written informed consent of both the head neonatologist and head nurse was needed for the recruitment of the NICU. The participation in the study was voluntary, and withdrawal of consent was possible at any time.

### 2.2 Data collection

After consent from both the head neonatologist and head nurse, 86 NICUs were included in the study. Each head neonatologist and head nurse received a personalized and pseudonymized questionnaire. Moreover, anonymous staff questionnaires and self‐addressed reply envelopes were sent to the head neonatologist and head nurse, with the request to distribute them to all staff members at a team meeting. All staff nurses and staff physicians who worked at least 50% of a full‐time equivalent in the NICU were eligible to participate in the study.

A total of 1406 nurses and 496 physicians from 84 different NICUs returned their questionnaires to a data trust unit. The response rate was 52.5% for the nurses and 55.6% for the physicians, based on the team sizes that were reported by the head neonatologist and head nurse, respectively. However, only NICUs from which both the head neonatologist and the corresponding NICU staff data were available could be considered. For example, if we received questionnaires from the NICU staff, but no questionnaire from the corresponding head neonatologist, or vice versa, that NICU had to be excluded. This led to a final data set of 468 physicians, 1251 nurses, and 76 head neonatologists from 76 different NICUs.

### 2.3 Measurements

We were interested in the following individual and organizational characteristics.

***Existence of SOPs in general***

The head neonatologist and the NICU staff, nurses, and physicians, were asked whether written SOPs in general existed in their NICUs.

***Existence of SOPs for PM***

The head neonatologists of each NICU were asked whether written SOPs for PM existed in their NICUs.

***Application of SOPs for PM***

The NICU staff were asked whether written SOPs for PM were applied in their NICU's daily routine.

***Additional clinical training***

The NICU staff were asked to state whether they had completed additional clinical training. Nurses stated whether they had completed additional clinical training in the field of pediatric intensive care and/or anesthesia and intensive care; Physicians stated whether they were medical specialists and had completed additional clinical training in the field of neonatology or pediatric intensive care.

***Working experience***

The NICU staff were asked to state their working experience in their current NICU in years and months, respectively.

***Bed turnover rate***

We calculated the BTR as the average number of VLBW infants treated in 1 year per NICU bed. The number of NICU beds and the number of VLBW infants were stated by the head neonatologist in each NICU. In our study, we defined infants with a very low birth weight (VLBW) as those infants with a birth weight of less than 1250 g (<2.76 lbs).

***Perceived cooperative norms***

We used the five‐item scale developed by Chatman and Flynn,^27^ which measures the perceptions of cooperative norms of the NICU staff. The scale was translated into German on the basis of in‐depth discussions with a bilingual (German/English) psychologist with expertise in item translation. For example, one item asked whether “There is a high level of cooperation among the members of the medical and the nursing service.” Respondents rated their perception on a 7‐point Likert scale, ranging from 1 (completely disagree) to 7 (completely agree). The scale showed acceptable internal consistency (α = 0.78). An average value of all five items was calculated for each respondent on an individual level.

We further controlled for the primary job and the NICU's team size and measured these variables as follows:

***Primary job***

The NICU staff were asked where they primarily work: Only in their NICU; in their NICU and in the pediatric intensive care unit (PICU); or in their NICU and in other areas of pediatrics.

***Team size***

The NICU's head neonatologist and head nurse were asked to state the number of physicians and nurses working in their NICU, respectively.

## 3 Data analysis

For our analysis, we only included data from teams whose response rate met the requirement of a sampling ratio of >0.32, based on Dawson^28^ and Richter et al.^29^ Furthermore, only data from physicians and nurses from NICUs where the head neonatologist took part in our study were included, as the information on the existence of SOPs could only be gathered from the head neonatologist's questionnaire. Data based on the head nurses' questionnaires were not used for our analyses. For a sample selection, see the Figure S2.

Besides analyses to describe the existence and application of SOPs for PM in German NICUs, we calculated four logistic multilevel models. We considered a *p*‐value of less than 0.05 as statistically significant. These models allowed us to consider the hierarchical data structure, that is, the nurses' and physicians' affiliation with their NICU, while exploring individual and organizational factors that might be associated with the application of existing SOPs for PM.

The analyses were performed using STATA (StataCorp. 2021. *Stata Statistical Software: Release 17*. StataCorp LLC).

## 4 RESULTS

### 4.1 Descriptive results

Our final data set consisted of 468 physicians (63.8% female) and 1251 nurses (98.2% female) from 76 NICUs. 93.4% (*n* = 71) were NICUs in teaching hospitals; among these, 19.7% (*n* = 15) categorized themselves as university hospitals. The majority of NICUs, that is, 85.5% (*n* = 65), provided a level I type of neonatal care. The average team size was 44.7%, and 76.4% of NICU staff were nurses. 20.5% of physicians and 43.8% of nurses had additional clinical training. The average working experience in the current NICU was 3.3 years (physicians) and 11.7 years (nurses). For further details, see Table 1.

**TABLE 1:** Participants' characteristics

	NICU sample (*n* = 76)			NICUs with SOPs for PM (*n* = 54)		
	Valid *n*	*N*	%	Valid *n*	*n*	%
Ownership	75			54		
Public		41	54.7		29	53.7
Nonprofit		29	38.7		22	40.7
Private		5	6.7		3	5.6
Teaching hospital	76	71	93.4	54	50	92.6
Level I	76	65	85.5	54	48	88.9

	Valid *n*	Mean (SD) [median]	Min; max	Valid *n*	Mean (SD) [median]	Min; max
Team size (overall)	76	44.7 (15.08) [44.5]	10; 92	54	46.8 (15.40) [46]	15; 92
Team size: Physicians	76	10.5 (4.23) [10]	4; 24	54	11.4 (4.51) [10]	4; 24
Team size: Nurses	76	34.2 (13.23) [32.5]	5; 77	54	35.4 (13.56) [33.5]	11; 77
NICU beds	73	22.1 (11.10) [20]	6; 71	52	22.0 (10.27) [20]	6; 56
Bed turnover rate (BTR)	60	1.7 (1.36) [1.4]	0; 7.7	45	1.9 (1.48) [1.6]	0; 7.7
Share of team members with additional clinical training	76	0.372 (0.15) [0.368]	0; 0.8	54	0.381 (0.15) [0.359]	0.1; 0.8

	Physicians (*n* = 468)			Nurses (*n* = 1251)		
	Valid *n*	*N*	%	Valid *n*	*n*	%
Age (in years)	466			1238		
<25		0	0		131	10.6
25–34		265	56.9		396	32.0
35–44		149	32.0		295	23.8
45–54		38	8.2		328	26.5
55–64		12	2.6		88	7.1
>64		2	0.4		0	0.0
Female	466	296	63.8	1242	1219	98.2
Additional clinical training	468	96	20.5	1251	548	43.8
Primary job	459			1225		
NICU		72	15.7		419	34.2
NICU and PICU		198	43.1		700	57.1
NICU and other areas of Pediatrics		189	41.2		106	8.7

	Valid *n*	Mean (SD) [Median]	Min; Max	Valid *n*	Mean (SD) [Median]	Min; Max
Working experience *(in years)*	460	3.3 (4.76) [1.5]	0.1; 32	1213	11.7 (10.17) [8]	0.1; 43
Perceptions of cooperative norms	467	5.8 (0.82) [6]	1.5; 7	1246	5.3 (1.02) [5.4]	1.4; 7

In total, 71.1% (*n* = 54) of head neonatologists confirmed the existence of SOPs for PM. Among these 54 NICUs, 48.5% (*n* = 160) of the physicians and 53.7% (*n* = 447) of the nurses declared that these SOPs for PM were also applied in their daily routine. Overall, a Wilcoxon signed‐rank test revealed that, in each NICU in which written SOPs for PM existed, the average share of physicians and the average share of nurses who stated that the SOPs for PM were applied in their daily routine did not differ (*Z* = 0.150, *p* = 0.881). This means that nurses and physicians perceived existing SOPs for PM as being applied equally.

Interestingly, in these 54 NICUs with written SOPs for PM, 6.1% of physicians and 10.7% of nurses responded that they did not even know whether or not written SOPs *in general* existed in their NICU.

**FIGURE 1:**
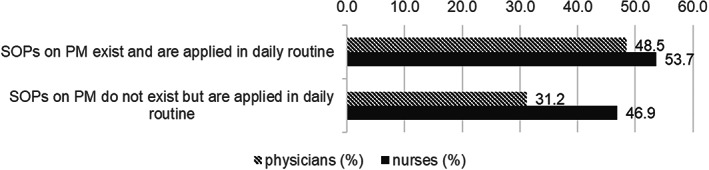
Application of SOPs for PM depending on the profession and existence

Further analyses revealed that 31.2% of physicians and 46.9% of nurses reported using SOPs for PM in their daily practice, despite their head neonatologist stating that such SOPs did not exist in the unit (see Figure 1).

### 4.2 Analyses

We were also interested in factors that were associated with the application of existing SOPs for PM.

For the 54 NICUs with SOPs for PM, we estimated four multilevel logistic models to identify factors influencing the perceived application of these SOPs for PM. Due to omissions on relevant variables, the multilevel logistic models were based on 44 NICUs with 277 physicians and 43 NICUs with 673 nurses.

The calculated intraclass coefficients (ICC) for the null models were 0.323 (considering only nurses) and 0.371 (considering only physicians). This means that 32.3% (37.1%) of the variance in the application of existing SOPs for PM by nurses (by physicians) could be explained by factors at hospital/NICU level.

We calculated four different multilevel logistic models separated by profession (nurses vs. physicians). Models 1 and 2 were based on the nurses' data, and Models 3 and 4 were based on the physicians' data. Models 1 and 3 only consisted of individual factors, whereas Models 2 and 4 also considered the NICU‐level organizational factors, such as the BTR and the team size of the own professional group, that is, nurses and physicians, respectively. The results are shown in Table 2.

**TABLE 2:** Results of multilevel logistic model

Multilevel logistic models|Dependent variable: Individual perception of the application of SOPs for PM								
	Nurses				Physicians			
	Model 1		Model 2		Model 3		Model 4	
	Odds ratio	*p*‐value	Odds ratio	*p*‐value	Odds ratio	*p*‐value	Odds ratio	*p*‐value
Additional clinical training	1.361	0.112	1.356	0.115	2.312	0.056	2.482^a^	0.037
Working experience in the current NICU	1.265^a^	0.014	1.261^a^	0.016	1.225	0.283	1.188	0.359
Primary job								
NICU and PICU	1.324	0.293	1.344	0.265	0.768	0.590	0.699	0.453
NICU and other areas of Pediatrics	0.637	0.275	0.651	0.298	1.274	0.647	1.078	0.885
Perceptions of cooperative norms	1.341^b^	0.005	1.322^b^	0.008	1.523^a^	0.015	1.507^a^	0.016
Bed turnover rate			0.649^a^	0.034			0.425^b^	0.004
Team size (own profession)			0.928	0.702			1.137	0.637
Constant	0.860	0.583	0.851	0.564	0.864	0.764	0.899	0.819
Number of observations	673		673		277		277	
Number of groups	44		44		43		43	
Log likelihood	−398.376		−396.195		−169.301		−164.728	
Wald χ^2^	18.29		22.69		13.42		19.70	
Prob > χ^2^	0.003		0.002		0.020		0.006	
AIC	810.753		810.390		352.604		347.456	
ICC	0.312		0.280		0.425		0.339	

^a^ *p* ≤ 0.05.

^b^ *p* ≤ 0.01.

Our analyses showed different results, depending on the profession.

We found additional clinical training to be a decisive factor that affected the perceived application of existing SOPs for PM—at least for NICU physicians in Model 4 (OR: 2.482, *p* ≤ 0.05, 95% CI: 1.055 to 5.842). We did not find that association in Models 1 and 2 when considering only nurses. Thus, Hypothesis 1, which stated that additional clinical training is positively associated with the application of existing SOPs for PM, was partially supported.

Hypothesis 2, which posited that professional experience in the current NICU is positively associated with the application of existing SOPs for PM, was also partially supported. Our results showed that professional experience was an important predictor for the application of SOPs for PM by nurses (OR: 1.265, *p* ≤ 0.05, 95% CI: 1.048 to 1.528). Analyzing the average marginal effects based on Model 1, it could be estimated that the probability of a nurse perceiving the existing SOPs for PM as applied increased by 0.44 percentage points per year of professional experience in the current NICU (*p* ≤ 0.05). A significant association between professional experience and the application of SOPs for PM was not found when only considering physicians.

Further, perceived cooperative norms were positively associated with the probability of SOPs for PM being applied. We found this positive association when considering only nurses (OR: 1.341, *p* ≤ 0.01, 95% CI: 1.090 to 1.650) and only physicians (OR: 1.523, *p* ≤ 0.05, 95% CI: 1.084 to 2.140), respectively. Consequently, Hypothesis 3, which posited that a high level of cooperation between physicians and nurses positively affects the application of existing SOPs for PM, was also supported.

Adding the BTR and the NICU's team size as organizational factors in Model 2 and Model 4, we found that a higher BTR decreased the probability of SOPs for PM being applied (Model 2: OR: 0.649, *p* ≤ 0.05, 95% CI: 0.436 to 0.967; and Model 4: OR: 0.425, *p* ≤ 0.01, 95% CI: 0.236 to 0.767). Consequently, Hypothesis 4—which stated that the higher the BTR of VLBW infants is, the lower is the application of existing SOPs for PM—was supported.

However, a log likelihood ratio test declared that there was no statistically significant difference between Models 1 and 2 ($\chi^{2}$(2) = 4.36, *p* = 0.113), that is, our second model, which also considered organizational factors and did not explain any better than Model 1 the variance regarding the application of SOPs for PM by nurses.

## 5 DISCUSSION

Nowadays, written SOPs for PM are common in most German NICUs. Nevertheless, our study revealed that the existence of PM strategies did not mean that these were directly applied in the daily clinical routine. Our results provided new insights for the field of implementation science. Overall, we found additional clinical training, working experience, and cooperative norms between nurses and physicians to be crucial factors that promoted the application of SOPs for PM. On the contrary, a high workload might reduce the likelihood of SOPs for PM being applied.

The call for guidelines addressing PM in NICUs, as well as their standardization at national level, has been reproduced in studies among NICUs in different countries.^11^, ^12^, ^14^ For the European region, Anand et al.^6^ reported that written guidelines for PM were available in 75% of the 243 recruited NICUs. Our study revealed a similar finding for the German NICUs, with about 70% of head neonatologists reporting the existence of SOPs for PM.

In NICUs with written SOPs for PM, only half of the physicians and nurses reported using these SOPs in their daily routine. This result highlighted that the mere existence of written SOPs did not lead to the application of these SOPs (see, for example,^30^, ^31^). Reasons why these SOPs were not applied could be manifold. Based on our multilevel model approach, we found various factors that influenced the probability of NICU staff perceiving the existing SOPs for PM as applied.

Our results underlined the importance of *clinical training* concerning the application of PM. We found that additional clinical training was important for physicians to perceive SOPs for PM as applied. Blomqvist et al.^32^ showed that fewer than half of the NICU physicians reported having sufficient knowledge of pain assessment using pain assessment instruments. This might lead to undertreatment, and thus, to fewer infants experiencing adequate PM. Further, Carlsen Misic et al.^19^ found out that physicians sometimes feared negative side effects of a pharmacological treatment and were not willing to prescribe a specific treatment. Thus, it could be suggested that physicians with additional training in neonatology and pediatric intensive care (a) felt more familiar with pain assessment instruments and (b) feared the negative side effects of a prescribed treatment less often, as the particularities of pharmacotherapy were part of the additional training. Such training for PM has been reported to be feasible and effective.^33^ Our findings indicated that clinical training might be a great contributor in ensuring that pain was adequately identified and treated. Thus, it could be suggested that pain assessment and management become part of the basic NICU training for all NICU staff.

In previous research, nurses named insecurity and perceived insufficient knowledge as barriers to using pain scales and managing patient pain.^19^, ^34^ Our results showed that nurses with longer *working experience* applied SOPs for PM more often in their clinical practice than nurses with less working experience. This result is consistent with the findings of Rolfe et al.^24^ They showed that nurses used their own clinical experience *beside* national guidelines and local policies as an important influencing factor on their clinical practice. However, the results of our study suggested that experience based on clinical training and working experience might be a *prerequisite* for adopting guidelines and policies and finally implementing them. Nevertheless, more research will have to be performed to verify this suggestion. Furthermore, our results might indicate that for newly graduated nurses it could be more challenging to understand and apply SOPs due to their lack of working experience. Widarsson et al.^35^ stated that newly graduated nurses reported difficulties translating their theoretical knowledge into practice. Another reason could be that new nurses did not know where to look up these written SOPs.

For nurses, working experience in their NICU might have improved their standing with NICU physicians and been more relevant than additional clinical training. Prior research reported that nurses noted that physicians sometimes disagreed with their pain assessment.^19^ The interprofessional collaboration between nurses and physicians was more challenging for new nurses with less working experience than for nurses with more working experience.^36^ However, clinical training may also be important for nurses to increase the application of SOPs.^16^, ^19^, ^22^, ^34^

In general, we did not find any difference between the physicians' and the nurses' assessment regarding the application of SOPs for PM. In NICUs with written SOPs for PM, physicians and nurses reported equally that these SOPs were applied. However, Tylor et al.^17^ showed that nurses more often used standardized pain scales to assess pain effectively. Nonetheless, frequently only the physicians' pain assessment was decisive for the actual analgesic use.^17^ This discrepancy might lead to NICU staff perceiving SOPs for PM as not being fully applied. Our results showed a positive association between *perceived cooperative norms between nurses and physicians* and the application of SOPs for PM. Thus, good interprofessional collaboration might increase the application of SOPs for PM. This positive effect could be explained by better communication between nurses and physicians, as well as by a clear and accepted distribution of roles. These aspects might help overcome a lack of professional autonomy and a feeling of powerlessness, which have been known as barriers to PM.^34^

Another finding to emerge from our analysis was that a higher *BTR* might have negatively affected the application of SOPs for PM. We focused on the BTR as an extended measure for a NICU's utilization. This measure allowed us to take the numbers of admissions and discharges into account. In general, the more different patients are treated in a shorter time period, the more complex the required care becomes, as providers have to adapt to new patients more often. Considering the complexity, we focused on infants with a VLBW, who experienced a higher risk of mortality (for example, see the study by Draper et al.^26^). The negative association between a higher BTR and the application of SOPs for PM may be related to two different aspects.

First, it can be assumed that the higher the BTR is, the higher the workload is in the NICU. This higher workload may cause less time for the NICU staff to deal with guidelines like SOPs and consult with colleagues on these guidelines.^24^ For that reason, NICU staff should be given sufficient time to become familiar with the SOPs.

Second, like all newborn infants, VLBW infants are also unable to verbalize their pain and thus, for example, to name the intensity or localization. Nevertheless, they communicate their pain by behavioral pain‐signaling, for example, crying, body movements, and facial expressions.^37^ Yet, prior research has described the restricted ability of children to cooperate and communicate as a barrier to pain relief methods by nurses.^23^, ^25^ This might be especially true for VLBW infants. Previous studies reported that VLBW infants might be less able to show their pain, for example, through facial activities.^38^, ^39^ NICUs with a higher BTR treat relatively more VLBW infants than NICUs with a lower BTR. At the same time, the existing SOPs for PM in these NICUs might possibly be less applicable for assessing and managing the pain of VLBW infants, rather than that of other preterm infants. Based on that, it can be suggested that SOPs for PM should also consider the infant's gestational age, and all health care professionals should be trained to recognize pain signals, especially those of VLBW infants.

Additionally, further research should investigate whether the adoption of SOPs for PM possibly requires a continuity between the NICU staff and the VLBW infants. In NICUs with a higher BTR, the NICU staff has to adapt to new patients more often. It might be possible that the interpretation of children's behavioral pain‐signaling succeeds better if these children are known longer.

### 5.1 Limitations

Selection and nonresponse bias might limit the validity of this study. For instance, because of the small number of recruited private NICUs, we were not able to analyze NICU ownership. Possibly, the NICU's ownership as an organizational factor may have influenced the findings. The number of university hospitals, of level‐I NICUs, and of NICU beds was also slightly higher among the recruited units. Therefore, our results may be of particular relevance to NICUs that predominantly treat very small and critically ill preterm infants.

In the framework of our questionnaires, the SOP was literally called “SOPs on pain *and stress* management.” However, we evaluated these SOPs as SOPs for PM only. Additionally, the head neonatologist as well as the nurses and physicians could only tick whether SOPs were available in general and, if so, which SOPs were applied in the daily routine. There were no free text spaces. Therefore, neither the head neonatologists nor the nurses or physicians could make any further comments. Hence, we had no information on, for example, their attitudes, pain scales used, or the reasons why certain SOPs were not applied in daily routine. Further, it would also be interesting to analyze how the application of SOPs develops over time, that is, whether new SOPs need time to be perceived as applied, compared with SOPs that exist for a longer time.

Surprisingly, we found a relevant number of NICU staff (about one‐third of physicians and half of nurses) who reported the application of SOPs for PM, although, according to the head neonatologist, no written SOPs for PM existed. Indeed, PM is often a topic included in written—but rather unofficial—induction concepts to train newcomers. Nurses and physicians might perceive these unofficial instructions as SOPs. Another reason might be that the head neonatologists were not aware of these SOPs, and there were SOPs for PM after all. However, if this was the case, the results from these NICUs would also highlight that more than half of physicians and nurses did not perceive existing SOPs for PM as applied in their daily routine.

The data were collected between 2015 and 2016. Thus, the number of NICUs with existing SOPs for PM and the perceived application of these could be different today. Further, although we are not aware of any new regulatory changes after this study was conducted that require the existence of SOPs for PM, changes in clinical practice may still have occurred, influenced by the scientific development in the field.

## 6 CONCLUSION

Although SOPs for PM are common in German NICUs, they are underutilized in daily clinical practice. However, staff in NICUs with written SOPs reported using PM strategies more often than staff in NICUs without these written SOPs. This study highlighted that—beside working experience—the clinical training to recognize, assess, and also to treat pain was essential. Furthermore, cooperation between nurses and physicians was important, as was sufficient time in the daily clinic routine, in dealing with these SOPs.

## FUNDING INFORMATION

This research project was supported by a grant from the UoC Forum “Managerial Risk Factors in Medicine” (Funding period: 2014–2016), which was funded by the Excellence Initiative Program at the University of Cologne, endowed by the German Research Foundation.

## CONFLICT OF INTERESTS

The authors have no conflicts of interest to declare.

## Supporting information


Figure S2
