# Supplementary figures and images for: Existence and perceived application of pain management protocols in German neonatal intensive care units
Source: Paediatr Neonatal Pain. 2022 Oct 5;4(4):149–57. doi: 10.1002/pne2.12089 (PMC9798041; doi:10.1002/pne2.12089)

Supporting Information


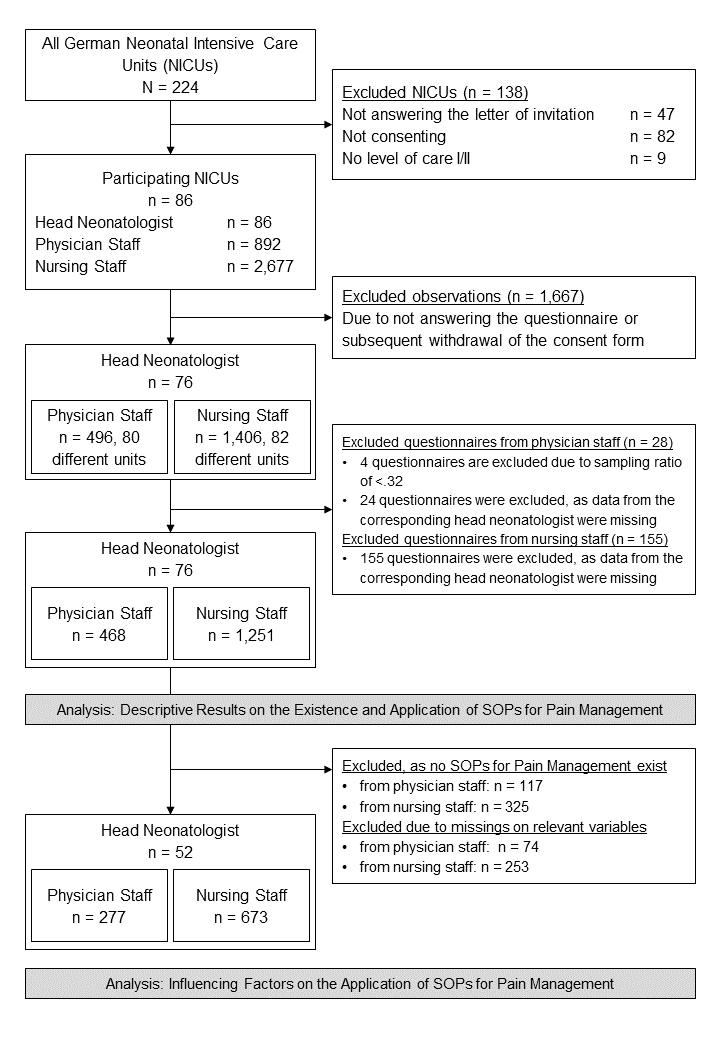


Figure 2 Sample Selection

Supplement: Supplementary file 1 — Figure S2 [file PNE2-4-149-s001.docx]
